# Imaging effects of hyperosmolality on individual tricellular junctions†
†Electronic supplementary information (ESI) available. See DOI: 10.1039/c9sc05114g


**DOI:** 10.1039/c9sc05114g

**Published:** 2019-12-11

**Authors:** Kaixiang Huang, Lushan Zhou, Kristen Alanis, Jianghui Hou, Lane A. Baker

**Affiliations:** a Department of Chemistry , Indiana University , 800 E. Kirkwood Avenue , Bloomington , Indiana 47405 , USA . Email: lanbaker@indiana.edu; b Renal Division , Washington University Medical School , 660 S. Euclid Avenue , St. Louis , Missouri 63110 , USA

## Abstract

The use of hyperosmolar agents (osmotherapy) has been a major treatment for intracranial hypertension, which occurs frequently in brain diseases or trauma. However, side-effects of osmotherapy on the brain, especially on the blood–brain barrier (BBB) are still not fully understood. Hyperosmolar conditions, termed hyperosmolality here, are known to transiently disrupt the tight junctions (TJs) at the endothelium of the BBB resulting in loss of BBB function. Present techniques for evaluation of BBB transport typically reveal aggregated responses from the entirety of BBB transport components, with little or no opportunity to evaluate heterogeneity present in the system. In this study, we utilized potentiometric-scanning ion conductance microscopy (P-SICM) to acquire nanometer-scale conductance maps of Madin–Darby Canine Kidney strain II (MDCKII) cells under hyperosmolality, from which two types of TJs, bicellular tight junctions (bTJs) and tricellular tight junctions (tTJs), can be visualized and differentiated. We discovered that hyperosmolality leads to increased conductance at tTJs without significant alteration in conductance at bTJs. To quantify this effect, an automated computer vision algorithm was designed to extract and calculate conductance components at both tTJs and bTJs. Additionally, lowering Ca^2+^ concentration in the bath facilitates tTJ disruption under hyperosmolality. Strengthening tTJ structure by overexpressing immunoglobulin-like domain-containing receptor 1 (ILDR1) protein abrogates the effect of hyperosmolality. We posit that osmotic stress physically disrupts tTJ structure, as evidenced by super-resolution microscopy. Findings from this study not only provide a high-resolution view of TJ structure and function, but also can inform current osmotherapy and drug delivery strategies for brain diseases.

## Abstract 2

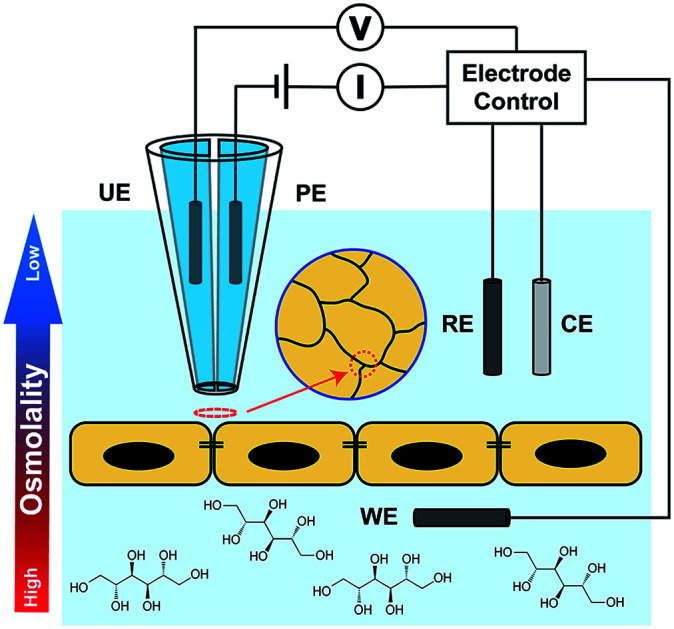
A nanoscale electrochemical imaging method was used to reveal heterogeneity present in conductance at epithelial cell junctions under hyperosmotic stress.

## Introduction

**Fig. 1:**
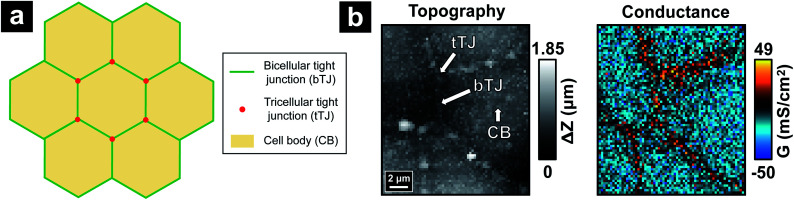
(a) Diagram of a hexagonal model of endothelial cell monolayer showing the organization of tricellular, bicellular junctions (tTJs, bTJs) and cell bodies (CBs). (b) Exemplified topography and corresponding conductance maps acquired by P-SICM on MDCKII cells. tTJs, bTJs and CBs can be identified in both images.

Intracranial hypertension (IH) is a significant complication frequently encountered in the practice of neurological care and can be life-threatening. IH can occur from almost all acute brain diseases or brain traumas, including cerebral edema, stroke, concussion and brain tumors.^1^–^4^ Osmotherapy has been the cornerstone of medical treatments for IH for decades and utilizes hyperosmolar agents including mannitol and hypertonic saline to create an osmotic gradient between the blood and brain. The gradient results in release of excessive brain fluid caused by IH to the blood and alleviates brain swelling.^5^,^6^ While effective, osmotherapy treatments are carried out through largely empirical practice based on experience, with adverse effects on the brain still unresolved.^7^ A major concern is the role of hyperosmolality on function of the blood–brain barrier (BBB), the protective and selective barrier between the blood and brain.^2^,^8^ Previous studies have shown that the addition of hyperosmolar agents can reversibly open tight junctions (TJs) at cerebrovascular endothelium of the BBB.^9^–^11^ TJs are multiprotein complexes accumulated at cell–cell junctions from which the integrity of the BBB is achieved.^12^–^14^ Thus, a comprehensive understanding of the effects of hyperosmolar treatments requires measurement at the single cell–cell junction or at subcellular structures. Presently, the overall barrier properties from TJs are typically characterized from bicellular tight junctions (bTJs) formed at the interface of two adjacent cells, and to a lesser extent, tricellular tight junctions (tTJs) where three or more cells meet^15^ (Fig. 1a). The structure and protein constituents of tTJs are completely different than bTJs, and tTJs have been shown to be integral components for the maintenance of barrier properties in endothelial tissue.^16^–^18^ However, because of the relatively low population of tTJs compared to bTJs and difficulties in separating out the contributions to transport from each type of TJs, tTJs remain largely unexplored. In addition – to our knowledge – the heterogeneity in the response of TJs (bTJ and tTJ) to hyperosmolality, let alone the response of individual tTJs, has not been previously reported.

Herein, we report the use of potentiometric-scanning ion conductance microscopy (P-SICM)^19^ to provide nanoscale analysis of the ion transport across Madin–Darby Canine Kidney Strain II (MDCKII) cells, a well-studied epithelial cell line that has been used as a model for the BBB,^20^,^21^ under the hyperosmolar condition regulated by the addition of mannitol. P-SICM is an advanced version of scanning ion conductance microscopy (SICM),^22^ a non-contact scanning probe technique particularly suitable for live-cell imaging. It utilizes a dual-barrel nanopipette as the probe to achieve both SICM imaging and potential measurement from which local conductance at the probed position can be acquired. This conductance value can be represented as the reciprocal of local transendothelial/transepithelial electrical resistance (TEER). Precise probe control at the nanoscale also endows P-SICM with the ability to differentiate the local conductance at cell bodies (CBs), bTJs and tTJs.^19^,^23^ Recently, we further combined hopping mode technique^24^ with P-SICM to allow simultaneous topography and conductance mapping.^25^ Ion transport properties of a whole cell area can be visualized dynamically, with bTJs and tTJs clearly resolved (Fig. 1b).

Specifically, in this study we compared the conductance maps of MDCKII cell monolayers before and after the addition of mannitol to the basolateral side so that the alteration of CBs, bTJs and tTJs can be observed. To quantify the effect of hyperosmolality on individual tTJs and bTJs, we designed an algorithm using computer vision to extract corresponding pixels and then automatically calculate their averaged conductance and difference between two conductance maps. This algorithm can help to avoid human bias in data selection and can also provide insight into feature detection for scanning probe microscopy. We further studied factors influencing the hyperosmolar effect on tTJs including Ca^2+^ concentration in bath solution and overexpression of immunoglobulin-like domain-containing receptor 1 (ILDR1) protein in the cells. Physical delocalization of individual tTJs under hyperosmolality was corroborated with super-resolution fluorescence imaging.

## Results and discussion

### Measurement of bulk transepithelial resistance

We first investigated the bulk effect of hyperosmolality on the overall MDCKII cell monolayer by conducting standard TEER measurements as a point of reference. TEER of the cell monolayers was monitored for 90 minutes after basolateral addition of mannitol as shown in Fig. S1.† During the time course of the measurement, TEER of control samples showed a typical response with a generally stable resistance, which indicated that the barrier function of MDCKII cells was not disrupted in physiological and ambient conditions. As 100, 200 and 300 mM mannitol were introduced into basolateral side of the cells respectively, TEER dropped to ∼85% of original value within 10 minutes and sustained this same level for the rest of recording time. These results are in accordance with other TEER studies of epithelial cells, and show that hyperosmolar agents such as mannitol upset TJs and the effects are observed quickly.^26^–^29^ Cells survived conditions of osmotic stress and additional reduction in TEER was not observed. In addition, the TEER response was not dose dependent in the range of 100–300 mM mannitol. This suggests that for mannitol concentrations examined here, hyperosmolar effects on MDCKII cells become saturated in terms of large-scale measurements of TEER, and barrier disruption is stable after reaching the saturation point. Although TEER measurements such as these cannot show the individual behaviors of TJs, let alone any heterogeneity in the responses of bTJs and tTJs, such macroscopic measurements provide a straightforward evaluation of overall barrier function. These measurements are helpful for evaluating possible influence on ion permeability under conditions of hyperosmolality for MDCKII cells and narrows selection of the best working conditions for subsequent P-SICM conductance mapping.

### P-SICM conductance mapping reveals heterogeneity in TJ response to hyperosmolality

Apparent local conductance (*G*) values determined from P-SICM can be considered as the reciprocal of local TEER measurements.^19^,^30^ Apparent conductance can also be utilized to further calculate the permeability of TJs to specific ions, as demonstrated in previous studies.^31^ Hence, *G* values can represent the barrier properties of locally probed positions. When measured in an imaging fashion, the *G* value at each pixel reports the local conductivity in the area directly under the nanoscale pipette tip. Since probe control of P-SICM offers scanning with nanoscale resolution, the step distance between adjacent pixels (250 nm in both *x* and *y* directions here) is typically small enough to detect the characteristic responses from cell bodies, bTJs and even tTJs.

**Fig. 2:**
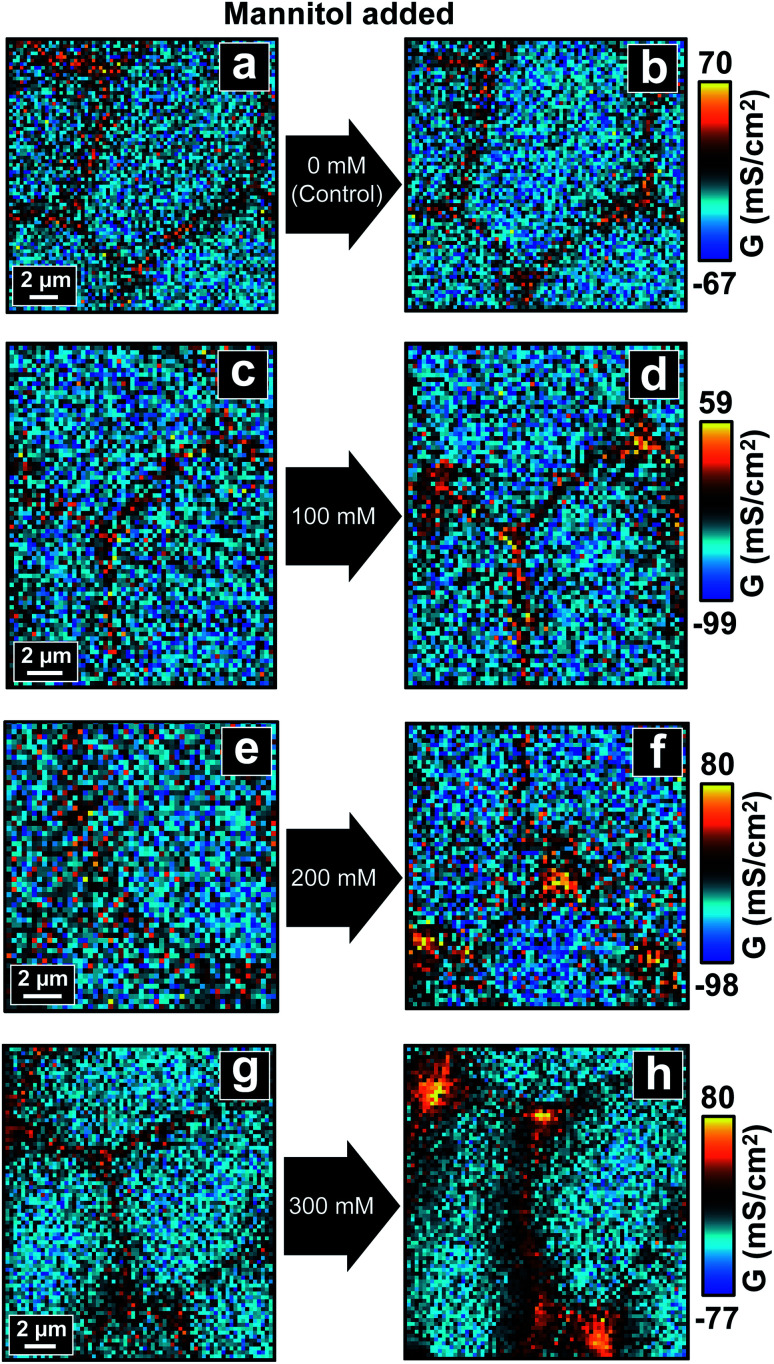
P-SICM conductance maps of MDCKII cells before (a, c, e and g) and after (b, d, f and h) mannitol was introduced into basolateral side.

To study effects of hyperosmolality on MDCKII cells with P-SICM, data for both topography and local conductance over the same cell area was acquired before and after the addition of mannitol in the basolateral compartment (Fig. 2). The scan time of each map was set ∼80 minutes, resulting in a total experimental duration of 3.5–4 hours after the cell sample was taken out of the incubator. To assure the cell viability and stability of TJs under such experimental duration, we also performed endurance tests where the MDCKII cells were scanned by P-SICM three times successively which took nearly 5 hours to finish (Fig. S5†). The integrity of MDCKII monolayer after test was evaluated by electrochemical impedance spectroscopy (EIS) as shown in Fig. S6.† Both bTJs and tTJs were found to be stable for at least 3 P-SICM scans, which is sufficient to support the 2 P-SICM scans required for studying the effect of hyperosmolality.

For control experiments, two consecutive P-SICM scans were performed on a sample area without any addition of mannitol, resulting in two conductance maps which typically displayed little to no obvious differences (Fig. 2a and b). This key control experiment further confirms the integrity and stability of MDCKII cell monolayers under P-SICM imaging conditions employed here. Additionally, the general appearance and magnitude of *G* values (*z*-scale) of all conductance maps before introducing hyperosmolar agents (Fig. 2c, e and g) are similar to those recorded for the control.

With addition of basolateral mannitol (Fig. 2d, f and h), conductance of transcellular pathways across CBs (*G*^CB^) stayed constant, suggesting that the cell membrane is able to withstand osmotic pressure conditions used here. In contrast, paracellular pathways across cell–cell junctions revealed remarkable differences between the responses at bTJs (*G*^bTJ^) and at tTJs (*G*^tTJ^). While bTJs did not show significant variance, tTJs lost barrier function and exhibited significant increases in local *G* values. Further, elevation of *G*^tTJ^ became more pronounced with increases in mannitol concentration to the basolateral chamber. Morphology of cells started to change due to cell volume adjustment resulted from osmotic gradient. As mannitol concentration reached 300 mM (Fig. 2h), bTJ regions also began to exhibit changes in conductance, suggesting larger scale junctional disorganization. In sum, we conclude that under conditions employed here, hyperosmolality depresses the barrier properties of MDCKII cell layers significantly at tTJs. To our knowledge, this is the first report to observe heterogeneous conductances between bTJs and tTJs under osmotic pressure, a finding that may have significant implications in tissue barrier function *in vivo*.

### Quantification of P-SICM conductance maps by computer vision

Since P-SICM can differentiate CB, bTJ and tTJ regions, the *G* value at a single cell, bTJ and tTJ in a P-SICM conductance map can be acquired by averaging pixels constituting corresponding areas respectively. Images treated in this manner can provide clearer and more comparative data demonstrating effects of hyperosmolality on different components of MDCKII cells, especially individual tTJs. We consider such data treatment as quantification of P-SICM conductance images. However, manually differentiating relevant pixels of a desired area (*i.e.* CB, bTJ and tTJ) is time-consuming and prone to unconscious human bias. To address this issue, we designed an automated algorithm utilizing computer vision to define and extract data from junctional areas in a P-SICM conductance map and then automatically calculate averaged *G* values to reveal local barrier properties of each CB, bTJ and tTJ region in the map.

Fig. S7† depicts an overview of the algorithm for detecting the junctional areas. The input of the algorithm program is a P-SICM conductance map in the form of a 2D matrix consisting of *G* values. First, the program coarsely distinguishes junctional areas from CBs based on the fact that *G* values of junctional areas are intrinsically higher than those of CBs.^19^,^30^ A threshold *G* value between those of CBs and junctional areas (*e.g.* average of the whole conductance map) is set by the user. All the pixels below the threshold are zeroed, and pixels above the threshold are scaled from 0 to 255. A grayscale uint8 image can thus be obtained, with CB regions primarily in black pixels and junctional areas in white or gray pixels. Noise reduction functions are then applied to remove incoherent non-zero pixels and smoothen the shape of non-zero areas. To have a clearer display of junctional areas, a skeletonization function^32^ is utilized to thin non-zero areas, which results in a topological skeleton outlining the center of junctional areas. The skeleton is pruned afterwards, followed by connections between nearest discrete neighbor pixels which form the final skeleton image with continuous pixels. The center of each tTJ area can thus be determined from the intersection points of the skeleton.

**Fig. 3:**
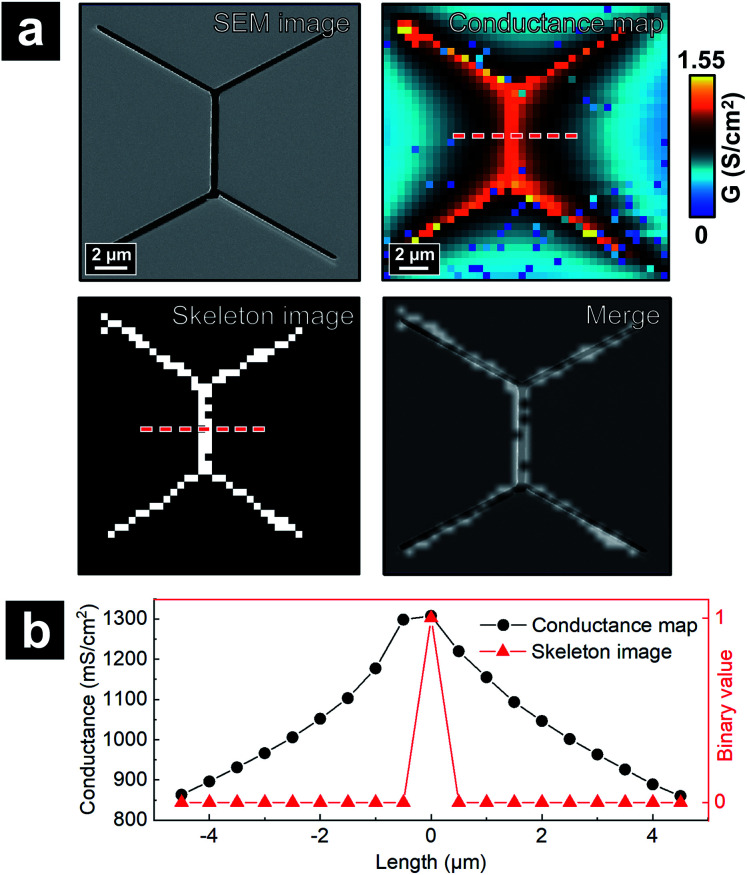
(a) Validation of computer vision algorithms with P-SICM conductance map of model junctions on Si_3_N_4_ membrane created by FIB. (b) Line profiles drawn from red dash lines in conductance map and skeleton image of (a) respectively.

To validate that the skeleton acquired from the program can accurately represent the position of junctional areas, we performed the algorithm on the P-SICM conductance map obtained from well-defined model cell junctions. The model cell junctions were created by focused ion beam (FIB) on a Si_3_N_4_ membrane, as shown in the scanning electron microscopy (SEM) image of Fig. 3a. In the corresponding conductance map, junctional areas penetrated by ion beam exhibited much higher *G* values than membrane, which serve as a model for MDCKII cells. The algorithm to acquire skeleton image successfully followed the feature of model junctions. As the skeleton was merged with the SEM image (Fig. 3a), tTJ regions of two images were well matched with minimal discrepancy at the end of each junction. Error was mainly caused by the low image resolution (40 × 40 pixels), which made it difficult to accurately present the orientation angle of junctions. Fig. 3b shows line profiles extracted from the same position of the skeleton image and conductance map. In the profile of the skeleton image, the only nonzero point corresponds to the apex of the profile from the conductance map. This further exhibits the ability of the algorithm to recognize junctional areas.

**Fig. 4:**
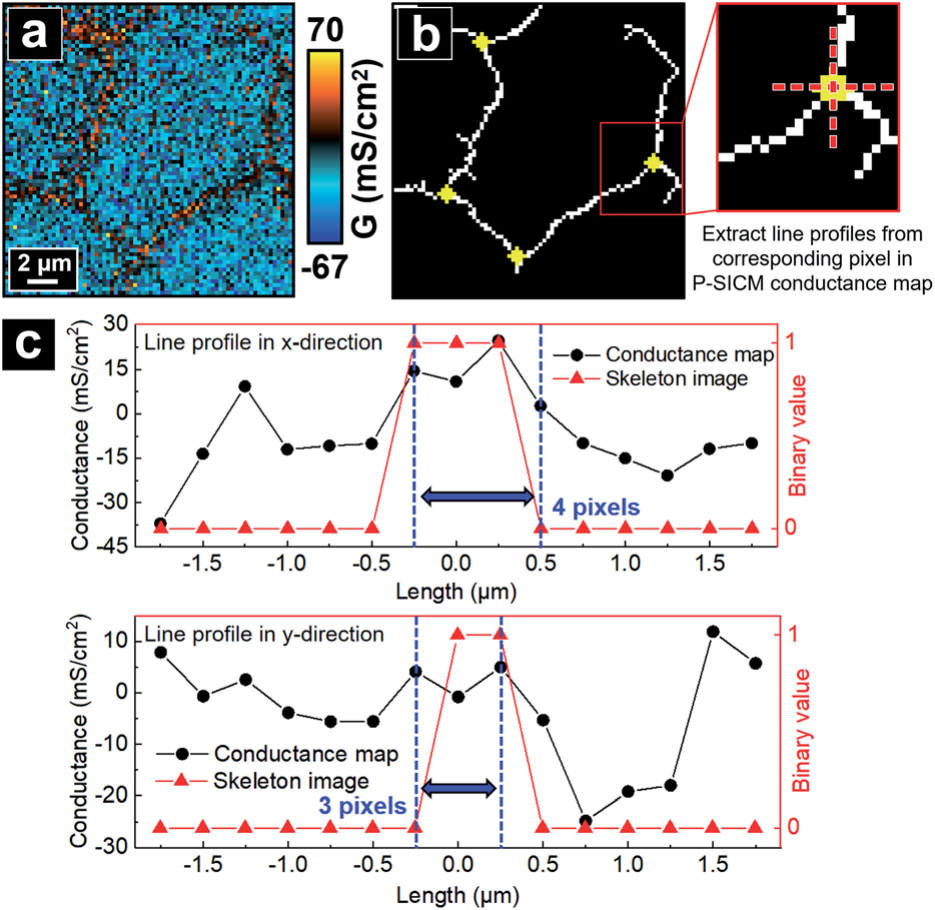
Schematic of the algorithm automatically calculating averaged *G* value of individual tTJ region. (a) A P-SICM conductance map used as an example. (b) Skeleton image obtained by the algorithm from (a), with tTJ points highlighted. (c) Line profiles drawn along the dash lines in (b) from skeleton image and corresponding pixels of P-SICM conductance map.

With the skeletonized image providing a map, the algorithm continues to determine the exact pixels making up each tTJ region respectively by matching pixels from the skeletonized image with pixels in the original data set. Fig. 4b shows an example of the skeleton image obtained from Fig. 4a, with tTJ center points highlighted. Each tTJ region is assumed to be circular in shape. To determine the diameter of each tTJ region, line profiles centering on each tTJ point (Fig. 4c) are drawn toward the *x* and *y* direction respectively in both the skeleton image (as shown in the right image of Fig. 4b which zooms in one of tTJ points) and corresponding positions in the conductance map. From the center of line profile (*i.e.* tTJ point), the program extracts the *G* values of neighboring points one after another toward both ends until it finds a point with a *G* value smaller than 5% of the center. The resulting two points on each side of the center are then marked as the boundary of tTJ region (indicated by two blue dash lines in Fig. 4c). The number of pixels between two boundary points is considered as the diameter of tTJ region. In reality, this region is larger than the physical tTJ, but the procedure used here isolates apparent conductance contributed from the tTJ. If line profiles from *x* and *y* directions give two different diameters, the smaller one is chosen as the final diameter to exclude possible influence from surroundings. Representative *G* values of each individual tTJ can then be calculated by averaging all pixels within the tTJ circle. In the line profile extracted from skeleton image, it is notable that all the points with value 1 (*i.e.* white pixel) lie between the boundary, which again demonstrates that the skeleton image can successfully follow the junction features of conductance map.

**Fig. 5:**
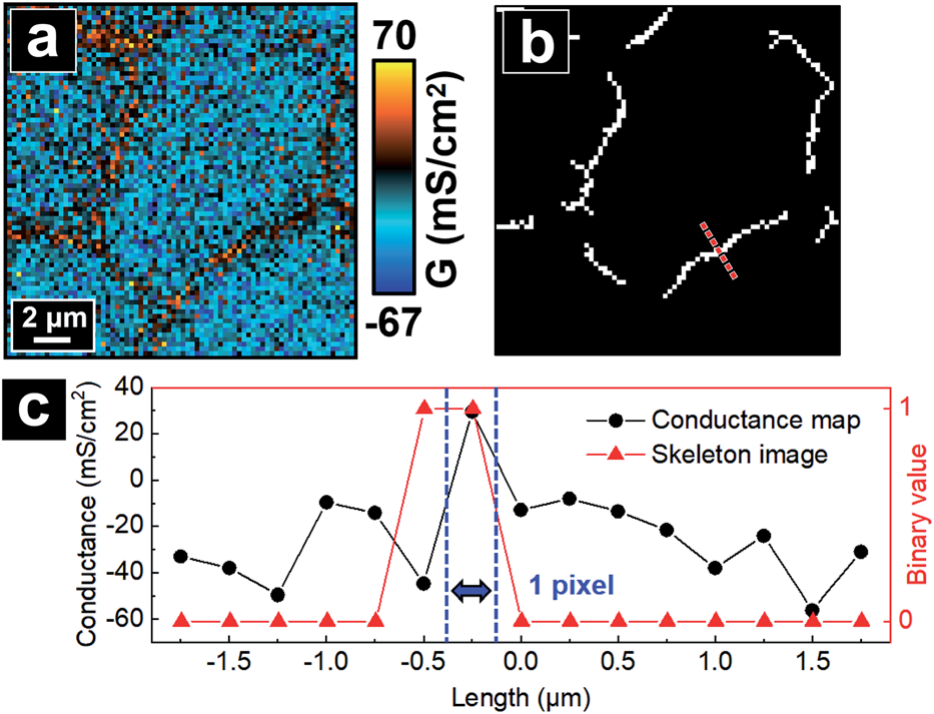
Schematic of the algorithm automatically calculating averaged *G* value of individual bTJ region. (a) A P-SICM conductance map used as example. (b) Skeleton image obtained from (a) with tTJ regions removed. (c) Line profiles drawn along the dash line in (b) from skeleton image and corresponding pixels of conductance map.

The algorithm next decides the pixels constituting bTJ regions. First, tTJ regions are removed from the skeleton image. To fully exclude the influence of tTJs on the calculation of *G*^bTJ^, these tTJ regions are expanded by adding 5 pixels to their original diameter in advance. Fig. 5b shows the resulted skeleton image after tTJ removal from Fig. 4b. The remaining branches formed by continuous white pixels can be regarded as the skeletons of bTJ areas. Exact pixels of each bTJ region are then determined from its thickness, which can be found by extracting line profile across its center. To obtain accurate line profile, the orientation of line drawn should be perpendicular to each bTJ skeleton, whose slope can be estimated from a Hough line transform. The same criteria for determining tTJ boundaries (see above) are used to find the two boundary points of bTJ (Fig. 5c). The thickness of bTJ can thus be defined as the number of pixels between these two points. Finally, the bTJ skeleton is dilated to have the same thickness and the resulting white pixels are used to match and calculate the *G* value of individual bTJs from the conductance map.

After *G* values of bTJs and tTJs are obtained, bTJ and tTJ pixels are both removed from the conductance map. The remaining pixels can be regarded as CB pixels and the *G* value of individual CB region can be calculated by averaging each divided piece consisting of continuous CB pixels. The final output of the program is a list showing the *G* values and standard deviations of every individual CBs, bTJs and tTJs. By inputting P-SICM conductance maps of the same cell area before and after mannitol treatment to the program, the effect of hyperosmolality on each cell component can be depicted by comparing its *G* value between the two maps.

**Fig. 6:**
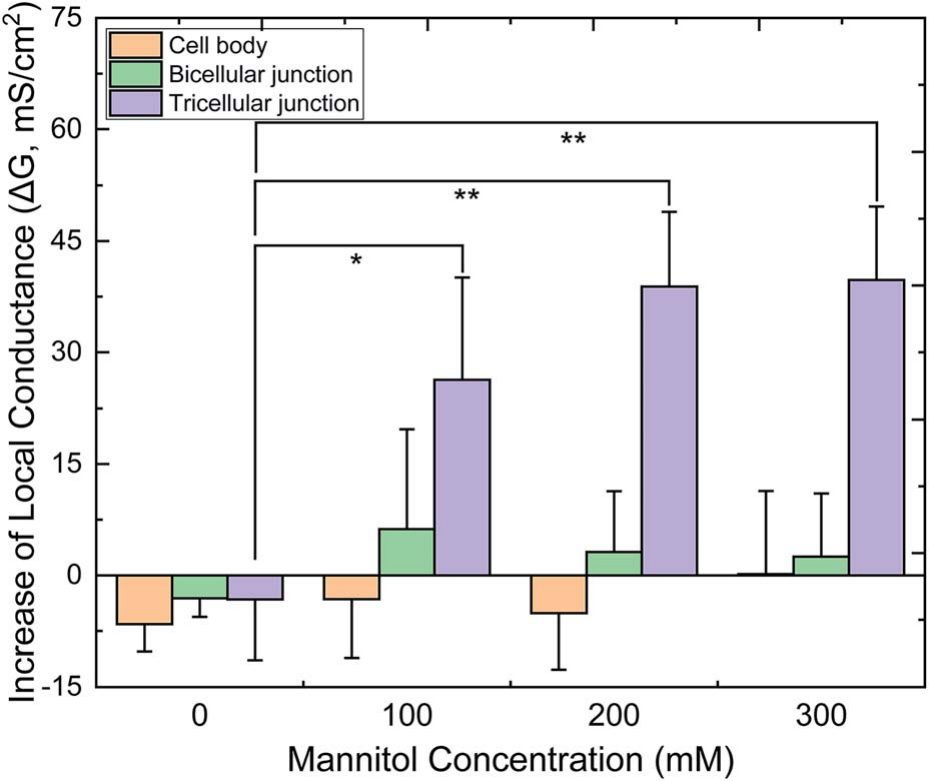
Quantified local conductance change (Δ*G*) of CB, bTJ and tTJ regions after basolateral mannitol treatment, as calculated by the algorithm. (*p < 0.05, **p < 0.01, *n* = 6–8 for each condition).

Fig. 6 displays the relative *G* values (Δ*G*) of CBs, bTJs and tTJs after basolateral addition of 0 (control), 100, 200 and 300 mM mannitol, as obtained by the automated program. To validate statistical analysis, multiple cell samples were studied for each condition to acquire data from sufficient number of individual CBs, bTJs and tTJs. Δ*G*^CB^ and Δ*G*^bTJ^ exhibited small variation less than ± 6 mS cm^–2^ in all conditions. On the contrary, Δ*G*^tTJ^ under hyperosmolality were statistically different from the control experiment, and *G*^tTJ^ could increase by 40 mS cm^–2^, which was 3-fold of its original values (13 mS cm^–2^ in average). This provides more explicit evidence proving that hyperosmolality mainly affects the barrier function of tTJs, and further demonstrates higher fragility of tTJ structure in dealing with extracellular osmotic stress. Interestingly, there was no significant difference between Δ*G*^tTJ^ under 200 and 300 mM mannitol. The effect of hyperosmolality on tTJs may reach saturation between 380–480 mOsmol per kg H_2_O, osmolality of the bath with 100–200 mM mannitol.

### The role of Ca^2+^ in tTJ opening under hyperosmolality

We next tested if the effect of hyperosmolality on tTJs can be adjusted by changing Ca^2+^ concentration in the bath solution. Ca^2+^ is known to play an important role in the formation of TJs and junctional adhesion molecules such as E-cadherin at cell–cell junctions.^33^,^34^ Depletion of Ca^2+^ in the cell buffer results in down-regulation of TJ proteins such as zonula occludens and the loss of TEER.^34^–^36^ In this study, Ca^2+^ concentration was lowered to ∼2 μM, a condition where barrier function may not be sustainable,^34^ and other ion components were kept identical. P-SICM measurements were then conducted with the same procedure as previously described except that milder hyperosmolality (25, 50 and 100 mM additional mannitol) was used to avoid conditions too harsh to keep the integrity of TJs.

**Fig. 7:**
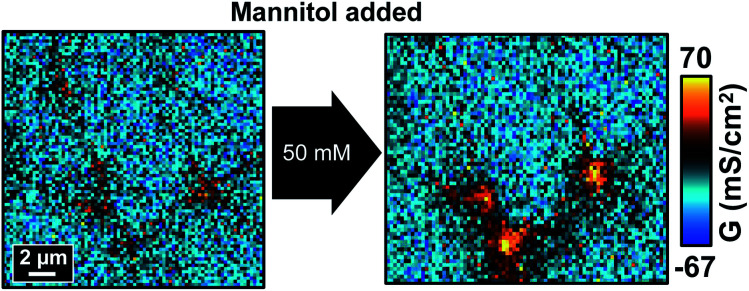
P-SICM conductance maps of MDCKII cells under low Ca^2+^ condition before (left) and after (right) 50 mM mannitol treatment.

As shown in Fig. 7 and S8a,† all the conductance maps before adding mannitol were similar to those scanned in normal Ca^2+^ concentration (Fig. 2), which suggested that the cell monolayer could still manage to maintain its barrier properties under 2 μM Ca^2+^. Furthermore, in control experiments the two consecutive P-SICM scans acquired almost identical graphs, revealing the stability of MDCKII cells to a greater extent and excluding possible influence from the duration of experiments. After the addition of mannitol, tTJs broke down as expected and showed remarkable increase in *G* values even when mannitol concentration was as low as 25 mM. *G*^CB^, *G*^bTJ^ and *G*^tTJ^ were further quantified by the automated algorithm from all conductance maps. Δ*G*^tTJ^ under control condition as well as under presence of 100 mM mannitol were compared to those obtained in normal Ca^2+^ concentration (Fig. S8b†). Statistical analysis indicated that lowering Ca^2+^ concentration could significantly aggravate tTJ disorganization. Surprisingly, bTJs did not show any loss of barrier function under these severe situations. It may be ascribed to the stronger structure of bTJ “strands”^13^ compared to tTJ “tubes”.^17^,^37^ Even though losing Ca^2+^ could loosen and disorganize some TJ components, the remaining bTJ components proved sufficient to resist osmotic pressure.

### ILDR1 strengthens tTJs to resist hyperosmolality

Previous experiments have demonstrated the high fragility of tTJ structure in comparison to bTJs. With this in mind, we then investigated the possibility of removing the influence of hyperosmolality by strengthening tTJ structure. One probable method to achieve this is to overexpress one of the proteins making up tTJ structure which would enable tTJs to seal more tightly. ILDR1, also known as angulin-2, has been shown to mainly localize at tTJs and is important for establishing tricellular contacts and tTJs formation.^38^–^40^ Wild type MDCKII cells do not express sufficient endogenous ILDR1 to be detected by common biological techniques such as immunoblotting and immunofluorescent labeling.^40^ To overexpress ILDR1 and enhance tTJ stability, MDCKII cells were transfected with *ildr*1 gene by retrovirus method to make homogeneous ILDR1 expression over the whole cell monolayer. The overall barrier properties of transfected cell line MDCKII-ILDR1 was evaluated by EIS. Fig. S9† shows representative EIS spectra of wild type MDCKII and MDCKII-ILDR1 cells. The resistance of ILDR1 cells was found to be almost 4-fold higher than wild type cells, indicating enhanced sealing of paracellular spaces after ILDR1 transfection.

**Fig. 8:**
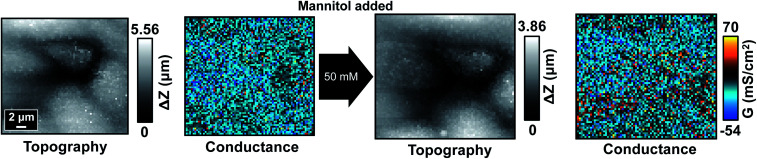
Topography and corresponding conductance maps of MDCKII-ILDR1 cells before (left) and after (right) 300 mM mannitol treatment.

P-SICM measurements were then carried out on ILDR1 cells in normal Ca^2+^ concentration and the mannitol dosage were restored to 100, 200 and 300 mM (Fig. 8 and S10†). Unexpectedly, in all conductance maps *G*^bTJ^ and *G*^tTJ^ decreased so enormously compared to wild type cells (Fig. 2) that paracellular transport could not be distinguished from transcellular regions. This suggested that inclusion of ILDR1 not only tightens tTJs but also bTJs. Since ILDR1 predominately accumulates at tTJs in epithelial cells, it must have interactions with other bTJ structure proteins, *e.g.* zonula occludens and claudins, to exert influences on the barrier properties of bTJs. A detailed mechanism of ILDR1 regulation on bTJs remains unclear and is the subject of further study.

After introducing mannitol, there were almost no changes in conductance maps even when 300 mM mannitol was present in basolateral bath, as shown in Fig. 8. Since junctional area cannot be identified from the conductance map, the automated algorithm could not extract its skeleton to obtain Δ*G* information. To address this limitation, for ILDR1 cells we modified the computer vision algorithm to acquire the skeleton image from their topography maps (Fig. S11†), based on the knowledge that cell–cell junctions are usually in lower topographical height than cell bodies. In general, the algorithm takes 2D height data matrix of topography map as input and finds local trough points from each row and column. A binary image with identical size of the topography map is then generated, where the determined trough points are set as 1 (white) and the rest of pixels are set as 0 (black). This image possesses similar appearance to uint8 image obtained from conductance map (see above) so that the following image processing steps described above can be applied to acquire the skeleton image. As shown in Fig. S11,† most of the white pixels in skeleton image can follow the junctional area in topography map. *G*^CB^, *G*^bTJ^ and *G*^tTJ^ can then be automatically calculated with the same method used for wild type cells.

Fig. S12† shows the quantification results of Δ*G*^tTJ^ of ILDR1 cells under different mannitol concentration in comparison to wild type cells. tTJs of ILDR1 cells all exhibited limited Δ*G* (less than 3 mS cm^–2^*versus* 40 mS cm^–2^ in wild type cells). Results indicate that the overexpression of ILDR1 can successfully improve the mechanical property of tTJs. Hence, we propose that hyperosmolality opens tTJs by physical osmotic stress instead of triggering chemical reactions that degrade tTJ structure (see below).

### tTJ protein delocalization under hyperosmolality

We hypothesize that the elevation of *G*^tTJ^ observed here under hyperosmolality results from disruption of the tTJ molecular architecture. The introduction of hyperosmolar agents triggers the release of fluid inside cells to achieve a balance against the osmotic gradient, which leads to a compensating adjustment of cell volume. This further exerts mechanical force on paracellular spaces including TJs between cells.^41^,^42^ As tTJs are multicellular contacts, they are more susceptible to these situations compared with bTJs.^17^,^43^ In addition, the pore size of tTJ “central tube” (10 nm)^37^ is much larger than bTJ channels (4–7 Å),^44^,^45^ hence the mechanical strength of tTJ structure against osmotic pressure may be weaker than that of bTJs. As a result, the possibility of breakdown for tTJs under hyperosmolality is much higher than that of bTJs, which can explain what we observed in P-SICM conductance maps.

**Fig. 9:**
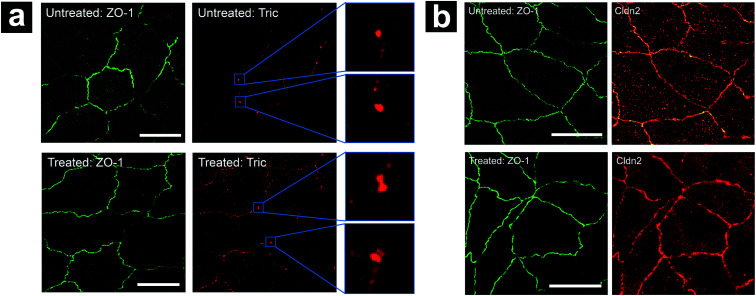
Effect of hyperosmolality on localization of (a) ZO-1 and tricellulin (Tric), (b) ZO-1 and claudin-2 (Cldn2). Scale bar in all images: 5 μm.

To further examine the status of tTJ and bTJ proteins under hyperosmolality, we immunolabeled zonula occludens-1 (ZO-1), tricellulin, and claudin-2 in MDCKII cells before and after 300 mM mannitol treatment respectively. Super-resolution structured illumination microscopy (SIM) images of labeled cells were obtained to provide fine details of tTJ and bTJ structures (Fig. 9). ZO-1 is a common TJ strand protein which can be used to depict the positions of cell–cell junctions as well as their barrier function.^46^,^47^ Tricellulin is another tTJ protein concentrated at tTJs and has been shown as a marker for maintenance of tTJ structural integrity.^15^,^18^ Claudin-2 is a TJ channel promoting the transport across paracellular spaces.^48^,^49^ As shown in Fig. 9a, tricellulin appeared as intact particles at tTJs under physiological condition. After the addition of mannitol, dumbbell-shaped features are present, which could be rationalized as tTJs split by the external force of cell volume changes, which agrees with our hypothesis. In contrast, there are no significant changes in the expression level of ZO-1 and claudin-2, suggesting that transport properties of bTJs were not affected which matches with P-SICM results.

## Conclusions

Results presented here are the first to discover the heterogeneity in the response of TJs to hyperosmolar treatment. Specifically, tTJs were found to be altered and show elevated local conductance under hyperosmolality, whereas bTJs and CBs did not exhibit obvious changes in barrier properties. This effect is dependent on the dose of hyperosmolar agents when the osmolality of the bath is relatively close to physiological condition (280 mOsmol per kg H_2_O) and becomes saturated at high osmolality. To further quantify observed effects, an automated computer vision algorithm was designed to extract the data of each individual tTJ and bTJ area from P-SICM conductance maps and calculated their averaged conductance values. Lowering Ca^2+^ concentration, which is important for TJ assembly, resulted in aggravated tTJ disruption. Overexpression of tTJ component protein ILDR1 in the cells can lead to opposite results, tightening cell junctions. The tTJ opening under hyperosmolality is hypothesized to result from delocalization of tTJ structure. Since tTJs are multicellular contacts, the influence of cell volume adjustment under osmotic pressure on tTJs is more pronounced than that on bTJs. While super-resolution fluorescence imaging cannot measure conductance changes observed with P-SICM, it can support conclusions made through immunolabeling of junctional proteins. This work provides super-resolution approaches to explore the function of TJ proteins and their interactions with extracellular conditions. Additionally, results suggest origins of possible adverse effects of osmotherapy on barrier tissues (including the BBB), and provides insights on the regulation of barrier function for the strategies of drug delivery.

## Conflicts of interest

There are no conflicts to declare.

## Supplementary Material

Supplementary information
