# Supplementary material for: Imaging effects of hyperosmolality on individual tricellular junctions
Source: Chem Sci. 2019 Dec 11;11(5):1307–15. doi: 10.1039/c9sc05114g (PMC7643560; doi:10.1039/c9sc05114g)
Supplement: Supplementary file 1 [file SC-011-C9SC05114G-s001.pdf]

Electronic Supplementary Material (ESI) for Chemical Science.
This journal is © The Royal Society of Chemistry 2019

Supplementary Information

Imaging Effects of Hyperosmolality on Individual Tricellular Junctions

Kaixiang Huang,[a] Lushan Zhou,[a] Kristen Alanis,[a] Jianghui Hou[b] and Lane A. Baker[*a]

[a.] Department of Chemistry, Indiana University, 800 E. Kirkwood Avenue, Bloomington, Indiana 47405, USA

[b.] Renal Division, Washington University Medical School, 660 S. Euclid Avenue, St. Louis, Missouri 63110, USA

***Corresponding author:**

Lane A. Baker

(812) 856-1873; (812) 856-8300 (fax);

E-mail: lanbaker@indiana.edu

Table of Contents：

1. Experimental section
2. Validation of the ability of P-SICM to reveal tight junction related properties
3. Long-duration control experiment as endurance test
4. Automated algorithm for quantification of P-SICM conductance map
5. The role of Ca^{2+} in hyperosmolar effect
6. The role of ILDR1 overexpression in hyperosmolar effect

S1. Experimental section

1. Chemicals and Materials

KCl (VWR International, LLC, Randor, PA), KH_2PO_4 (Mallinckrodt Baker, Inc., Phillipsburg, NJ), $NaHCO_3$ (Mallinckrodt Baker), NaCl (VWR International, LLC), Na_2HPO_4 (Mallinckrodt Baker), glucose (Sigma-Aldrich, Inc., St. Louis, MO), $CaCl_2·2H_2O$ (Mallinckrodt Baker) and $MgCl_2·6H_2O$ (Mallinckrodt Baker) were used to prepare Hank's balanced salt solution (HBSS, 138 mM NaCl, 5.3 mM KCl, 4.2 mM $NaHCO_3$, 0.34 mM Na_2HPO_4, 0.44 mM KH_2PO_4, 0.5 mM $CaCl_2$, 0.2 mM $MgCl_2$ and 5.6 mM glucose). Osmolality of HBSS was modified by addition of D-mannitol (Sigma-Aldrich) and confirmed with an osmometer (3300, Advanced Instrument, Norwood, MA). Electrolyte used for filling nanopipettes was 1× phosphate-buffered saline (PBS, Sigma-Aldrich). All aqueous solutions were prepared from Milli-Q water (resistivity = 18.2 MΩ·cm at 25 °C, Millipore Corp., Billerica, MA) and filtered with a 0.22 μm Stericup® (Millipore).

2. Cell Culture and Transfection

Madin-Darby Canine Kidney Strain II (MDCKII) cells were grown in Dulbecco's modified Eagle's medium (DMEM, D6429, Sigma-Aldrich) supplemented with 10% (v/v) fetal bovine serum (FBS, F4135, Sigma-Aldrich), 100 U/mL penicillin and 100 μg/mL streptomycin (P0781, Aldrich Sigma). Cells were maintained in T-flask at 37 ^{o}C in humidified air with 5% CO_2.

To obtain MDCKII cell lines homogeneously overexpressing ILDR1 protein (MDCKII-ILDR1), MDCKII cells were transfected with retrovirus method as previously described.[1] Briefly, the full-length mouse ILDR1 gene (NM_001285788) was cloned into the retroviral vector pQCXIN (Clontech Laboratories, Mountain View, CA). Molecular clones were verified by DNA sequencing. Vesicular stomatitis virus glycoprotein (VSV-G) pseudotyped retroviruses were produced in human embryonic

kidney cells 293 (HEK-293) and used to infect MDCKII cells at a titer of 10^6 cfu/mL. Resulted MDCKII-ILDR1 cells were maintained in the same condition as wild type cells.

Before P-SICM measurement (vide infra), cells in confluence were harvested with trypsin-EDTA (Mediatech, Tewksbury, MA) and seeded with 10^6 cells/cm^2 onto a home-made membrane insert. To prepare the insert, a piece of transparent polyethylene terephthalate (PET) membrane (pore size 0.4 µm, pore density 2×10^6 /cm^2, Corning, NY) was taped to the bottom of a petri dish with a hole in the center, and collagen (Sigma-Aldrich) of 150 µg/cm^2 was then added onto the membrane. Cell monolayers cultured on membrane inserts were maintained at 37 °C in a humidified air- 5% CO_2 atmosphere for 4-7 days to achieve confluence that displayed steady-state transepithelial electrical resistance (TEER).

3. TEER Measurements

To measure the overall structural integrity of tight junctions (TJs) in MDCKII cells, TEER value was measured with an endohm chamber (ENDOHM-24SNAP, World Precision Instrument, Sarasota, FL) connected to an EVOM2 epithelial voltohmmeter (World Precision Instrument). Cells were grown on a porous PET membrane insert (Corning) with 0.4 µm pore size and 4.2 cm^2 exposed culture area (*A*). The membrane insert with cells was placed in the endohm chamber which set one electrode at each side of the cell monolayer, and the resistance value (*R*) could be read from voltohmmeter. Background resistance of PET membrane (R_0) can be determined by the same fashion with a blank membrane insert. TEER value of the cell monolayer in $\Omega \times$ cm^2 was calculated as:

$$TEER = (R - R_0) \times A \tag{3.1}$$

Bulk Effect of hyperosmolality on MDCKII cell monolayer was studied by monitoring TEER over time. First, both sides of the cell layer were filled with HBSS

(osmolality = 280 mOsmol/kg H_2O), and TEER value was recorded continuously for 30 min to ensure that barrier properties was stable and in normal range. Bath in the basolateral side of cell layer was then replaced with HBSS supplemented with different amount of D-mannitol (0, 100, 200 and 300 mM with corresponding osmolality 280, 380, 480, 580 mOsmol/kg H_2O). TEER was recorded at the same time and further monitored for the next 90 min.

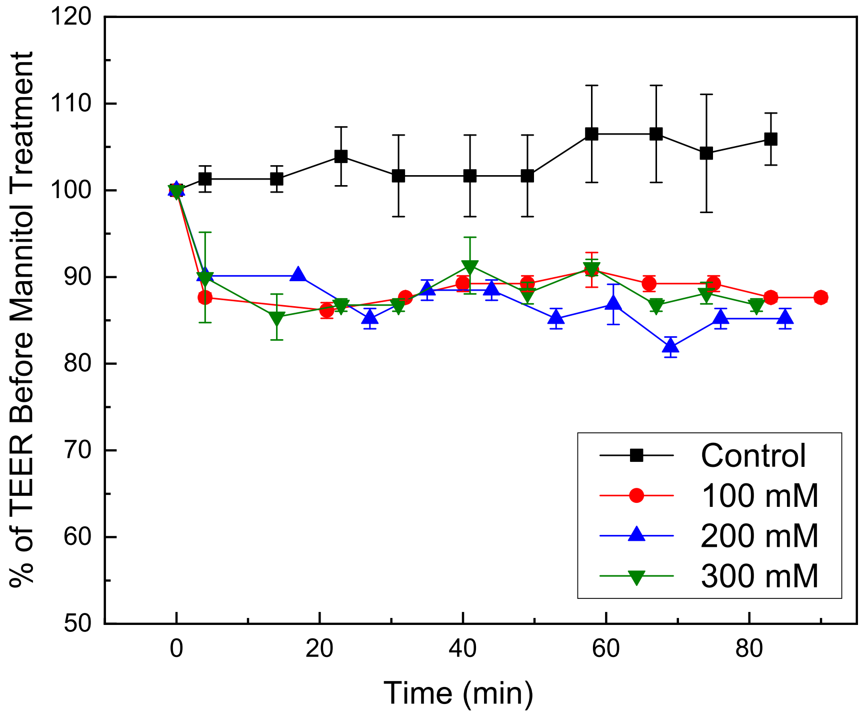

Figure S1. TEER changes of MDCKII cells after addition of mannitol in basolateral side. (n=3 for each condition)

4. Electrochemical Impedance Spectroscopy

Electrochemical impedance spectra of wild type MDCKII or MDCKII-ILDR1 cells grown on membrane insert were measured by a CHI 660C potentiostat (CH Instruments, Austin, TX) in three-electrode configuration. HBSS was filled in both sides

of cells. A peak-to-peak 10 mV sinusoidal wave with frequency ranged from 1 Hz to 100 kHz was applied to the cell monolayer, and the current response was recorded at 60 discrete frequencies. From the Nyquist plot obtained (Z_{Im} vs Z_{Re}), a semicircular response was observed and utilized to calculate the resistance of PET membrane and cell monolayer respectively.

5. P-SICM Setup and Measurement

P-SICM measurements (Fig. S2) were performed with a home-built SICM described previously.[2] Double-barrel nanopipettes for P-SICM scanning were pulled from quartz theta capillaries (QT120-90-7.5, Sutter Instrument, Novato, CA) with a CO_2 laser puller (P-2000, Sutter Instrument). Program used was as follows: *Heat=700, Fil=3, Vel=35, Del=160, Pul=160.* The pipette was characterized by scanning transmission electron microscopy (STEM), with inner diameter of each barrel determined as ~50 nm (Fig. S3). The nanopipette was then filled with PBS. Pipette electrode (PE, Ag/AgCl) was inserted in one barrel of the nanopipette and served for normal SICM feedback, while potential electrode (UE, Ag/AgCl) was placed in the other barrel to record local potential. The membrane insert bearing a live cell sample was mounted between two chambers of a perfusion cell, with both chambers filled with HBSS. Reference electrode (RE, Ag/AgCl) and counter electrode (CE, Pt) were placed in top chamber and held at ground potential with a customized electrode-control module. In the bottom chamber, another Ag/AgCl working electrode (WE) was placed to apply transmembrane potential across the sample.

To obtain the local conductance map, an FPGA board (Digilent, Inc., Pullman, WA) and its user interface were utilized to control the SICM feedback loop. Briefly, at the beginning and end of each "hop" during the hopping mode of SICM scanning, the feedback control was bypassed and paused for a preset duration during which a 4 V pulse was generated from the FPGA to trigger an external transmembrane potential. The transmembrane potential was applied to WE from an Agilent 33220A function

generator (Agilent Technologies, Santa Clara, CA) with respect to ground (RE and CE). Potentiometric signal at UE was recorded with a customized differential amplifier with 100× gain and 20 Hz low-pass filter. A digitizer (Axon Digidata 1440, Molecular Devices, Sunnyvale, CA) was used for monitoring the data from all scanning channels. The acquired channel data were collected by FPGA and the apparent local conductance (G) of the present probed position can be calculated as:[3]

$$G = \frac{E}{\rho V^e} = \frac{(\Delta V_{close} - \Delta V_{far})/\Delta z}{\rho V^e}$$

In this equation, *E* is the electric field between the two probe positions at the beginning and end of each "hop" when V_T is applied. *E* can be determined from dividing the difference of potential deflections at these two probe positions (ΔV_{close} - ΔV_{far}) by the hopping height (Δz). ρ is the specific resistivity of the bath solution in the top chamber. V^e represents the potential range of V_T (e.g. V^e = 100 mV if a ± 50 mV triangular wave is applied as V_T).

As the nanopipette scan over the sample to acquire topographical information, the local G value can also be simultaneously obtained. Thus, the conductance map can be generated after all pixels of a cell area is scanned.

For studying the effect of hyperosmolality, a P-SICM conductance map was first taken on the cell sample in normal condition (HBSS on both sides here), then the bath solution in the bottom chamber was changed to HBSS with defined concentration of mannitol and a second P-SICM scan was conducted on the same area. The effect can be visualized by comparison between two resulted conductance maps.

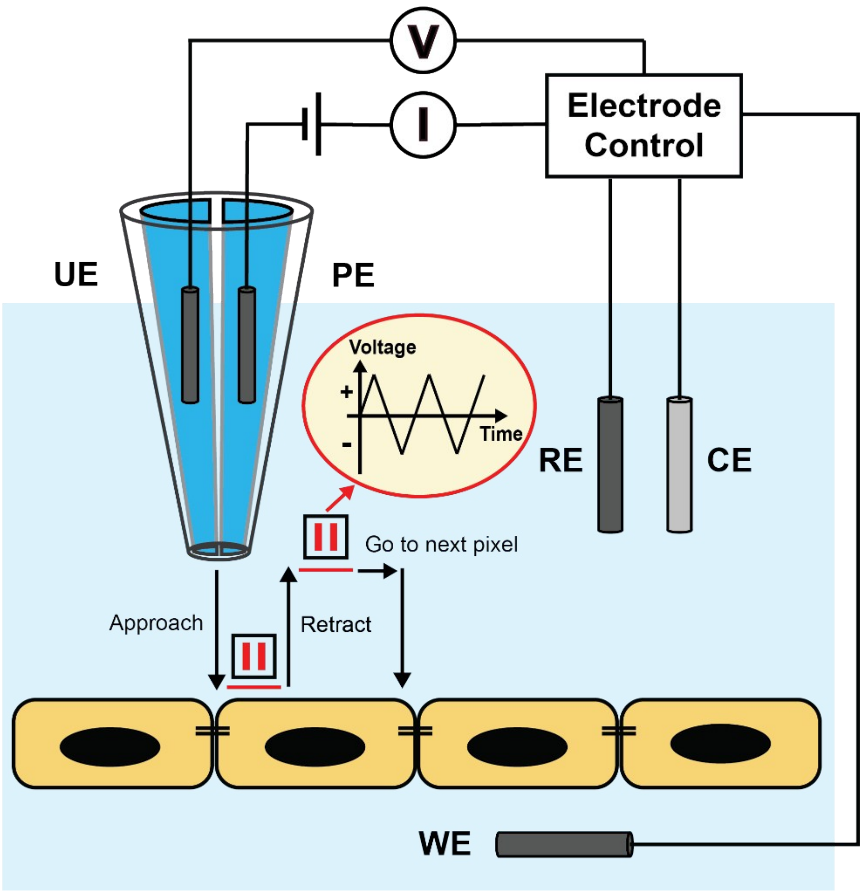

Figure S2. Configuration of P-SICM conductance mapping on cell monolayer.

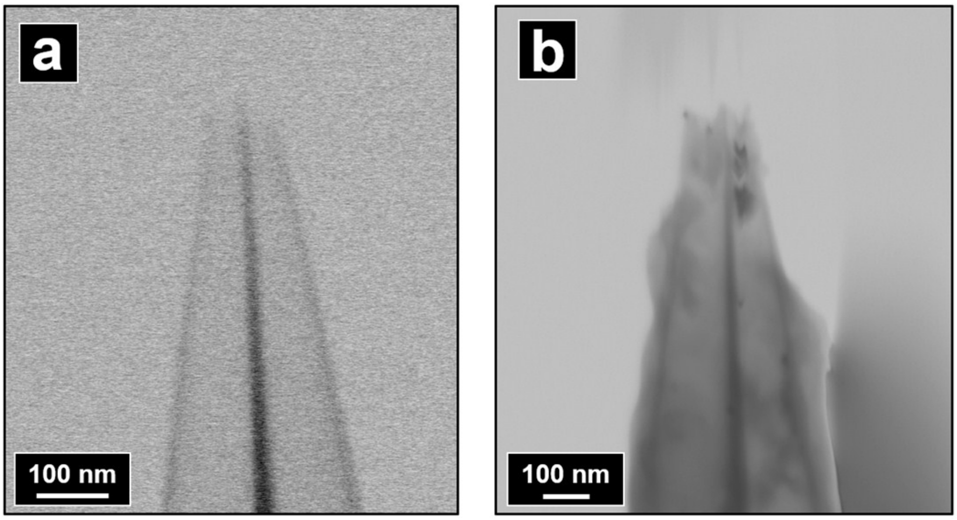

Figure S3. Scanning transmission electron microscopy (STEM) images of the P-SICM probe (a) before use and (b) after use.

6. Focus Ion Beam (FIB) for Model Cell Junction

The synthetic model junction for validation of computer vision algorithm (see below) was milled into a 50 nm thick Si_3N_4 window (Norcada Inc.) with a FIB instrument (Auriga 60, Carl Zeiss, GmbH) controlled by Nano-Pattering and Visualization Engine (NPVE; FIBICS, Inc.). The milling was performed with a 30 kV beam at 50 pA and a dosage of 0.160 nC/µm. Created junctions were further characterized by SEM of FIB instrument. Resulted Si_3N_4 window was then mounted on the perfusion cell and filled with HBSS on both sides for P-SICM measurements.

7. Immunolabeling of MDCKII Cells and Super-resolution Imaging

MDCKII Cells grown on PET membrane inserts were fixed with cold methanol at –20 °C, followed by blocking with PBS containing 10% FBS and incubation with primary antibodies (diluted 1:300) and FITC- or rhodamine-labeled secondary antibodies (diluted 1:200). After washing with PBS, slides were mounted with Mowiol (CalBiochem). Super-resolution images were collected with x100 oil immersion lens (NA: 1.40) by NIKON structured illumination microscopy (n-SIM). For the dual imaging of FITC and rhodamine, fluorescent images were collected by exciting the fluorophores at 488 nm (FITC) and 543 nm (rhodamine) with argon and HeNe lasers, respectively. All images were converted to TIFF format and arranged using Photoshop (Adobe).

8. Statistical Analysis

The automated algorithm output G values of each individual cell bodies (CBs), bicellular tight junctions (bTJs) and tricellular tight junctions (tTJs) present in the conductance map. Thus, the increased amount of G value (ΔG) for these features overall after mannitol treatment can be expressed as means ± error of the means. Statistical analysis was performed with Student's t-test. $p < 0.05$ was considered significant. Significance levels are denoted as *$p < 0.05$, **$p < 0.01$, ***$p < 0.001$. The number of features (i.e. CBs, bTJs and tTJs) measured is indicated by n.

S2. Validation of the ability of P-SICM to reveal tight junction related properties

To validate that P-SICM can reveal TJ-related functions or properties, P-SICM topography and conductance maps were compared to antibody stained Zonula occludens (ZO)-1 fluorescence images labeled to determine the location of tight junctions in the same area scanned. Topography (Figure S4a left), conductance (Figure S4a right) images of an area were first collected. After P-SICM scanning, the sample was fixed with cold methanol at –20 °C, followed by incubation with ZO-1 primary antibody conjugated with Alexa 488 (diluted 100:1). After washing with PBS, the sample was mounted on a glass slide, with fluorescence images collected by confocal microscopy (Leica SP8). Wide-field optical (Figure S4b) and fluorescence (Figure S4c) images were collected. The expanded region shown in Figure S4c indicates the location where P-SICM recordings were made. As shown in Figure S4d, when topographic or conductance images are layered with the fluorescence images, the location of TJs is collocated. From these images we also note that the topographic images are suitable for locating the position of cell body and cell junctions.

Figure S4. (a) Topography and corresponding P-SICM conductance maps of an area of MDCKII cells. (b) Optical image of the same area scanned indicated by the white dashed box. Scale bar: 25 μm. (c) Confocal fluorescence image of the same sample with tight junction protein ZO-1 labeled. The area scanned is indicated by the yellow dashed box and zoomed-in image on the right. Scale bar: 25 μm. (d) Merged images of zoomed-in fluorescence image from (c) with P-SICM topography and conductance maps from (a).

S3. Long-duration control experiments as endurance test

To assure the cell viability and stability of TJs under experiments 3.5-4 hours in duration, endurance tests were performed where the MDCKII cells were scanned by P-SICM three times successively (ca. 5 hours imaging (Fig. S5)). The integrity of the MDCKII monolayer before and after this test was evaluated by EIS as shown in Fig. S6. Both bTJs and tTJs were found to be stable for at least 3 P-SICM scans, which is strongly supports cell viability under conditions employed here (2 P-SICM scans) in examining the effects of hyperosmolality.

Figure S5. Three consecutive P-SICM conductance maps of the same area from MDCKII cells showing the time-evolution of TJs during the time course of P-SICM measurements. Both bTJs and tTJs were stable for at least 3 P-SICM scans. Frameshift in the images shown occurs due to instrumental drift.

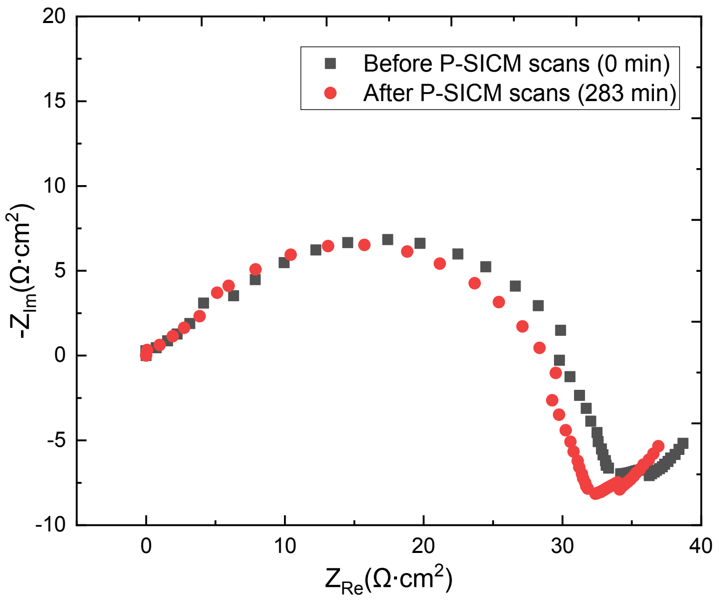

Figure S6. Impedance spectra of MDCKII cells before P-SICM scans (0 min, black) and after P-SICM scans (283 min, red).

S4. Automated algorithm for quantification of P-SICM conductance map

The automated algorithm for quantification of P-SICM conductance map was written in Python 3.7 supplemented with image processing module OpenCV (**cv2**). Fig. S7 shows the overview of extracting junction information. First, the data matrix of P-SICM conductance map is used as the input of the program. The averaged G value of the whole map is calculated and used as the threshold to zero all the pixels below it. The remaining pixels are then scaled to [0,255] which results in a grayscale uint8 image. To make the junction area more pronounced, before the scaling these remaining pixels can be multiplied by 255. If the pixel is larger than 255, its value is set as 255. The program next removes incoherent non-zero pixels defined as shapes with their number of continuous pixels (i.e. size) too small to be significant. **cv2.findContours()** function is utilized to find all the shapes present in the uint8 image as well as their number of pixels. Shapes smaller than the threshold size set by the users are eliminated. Median filter is then applied to remove other noises and smoothen the shape of non-zero areas. The image is further filtered by image thresholding which generates a binary image for the convenience of subsequent operations.

cv2.dilate() followed by **cv2.erode()** are used to eliminate small black pixels in the white objects (also referred to as "hole closing"). Next, skeletonization function[4] is used to extract the skeleton which outlines the medial axes of existing objects. The white pixels of preliminary skeleton image are usually not continuous, and thus the skeleton image is subjected to another set of noise removal functions and pruning. First, the same function of removing incoherent non-zero pixels is performed and the

skeleton images is dilated in advance to avoid excessive loss. “Hole closing” function is again applied to remove small holes in the shapes. Considering that the final skeleton of the junction areas should be a continuous object, a function named “gap closer” here was designed to predict and connect discrete shapes.

In “gap closer”, **cv2.findContours()** is first utilized to locate and count the shapes present in the image. These shapes are looped through to find their nearest neighbour and then connect with each other. The process is repeated until there is only one object recognized in the image.

After application of “gap closer”, the object is pruned by median filter and undergoes another skeletonization. “Gap closer” is conducted again to acquire the final skeleton image. tTJs can be located by finding the intersection points of the skeleton and further verified manually.

Once the skeleton image is obtained and tTJs points are selected, the program continues to calculate the G values of individual CB, bTJs and tTJs. The detailed procedures are introduced in the main text. The program outputs a list of calculated results. By comparing the results obtained from conductance maps before and after mannitol treatment, the increased amount of G value (ΔG) of CBs, bTJs and tTJs can be used to illustrate the effect of hyperosmolality.

Figure S7. Detailed steps of computer vision algorithm to obtain the skeleton of junctional areas from P-SICM conductance maps.

S5. The role of Ca^{2+} in hyperosmolar effect

To study the effect of Ca^{2+} on tTJ alteration, the sample was bathed in HBSS with ~ 2 μM Ca^{2+} and other ion components identical to original one. Mannitol dosages used here were 0 (control), 25 mM, 50 mM and 100 mM.

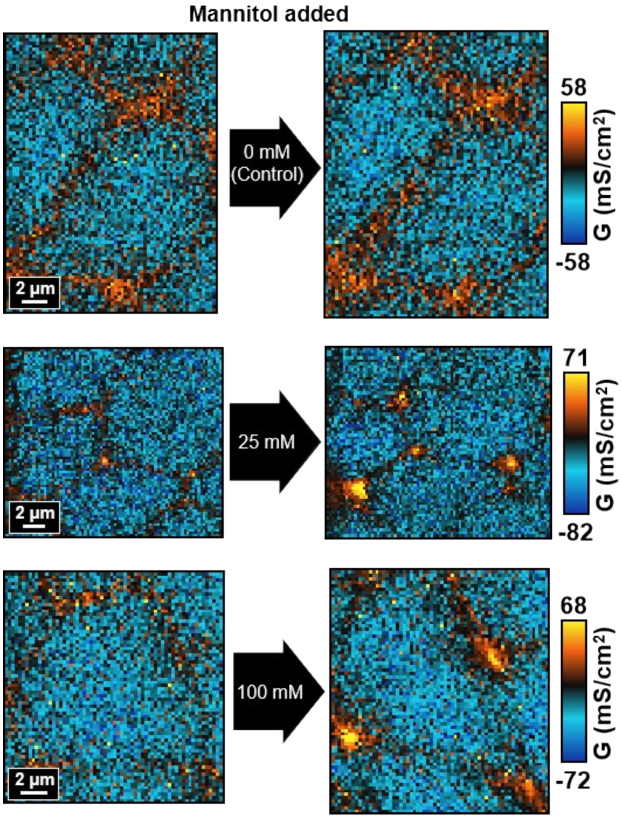

Figure S8a. Topography and corresponding conductance maps of MDCKII cells under low Ca^{2+} condition before (left) and after (right) mannitol treatment.

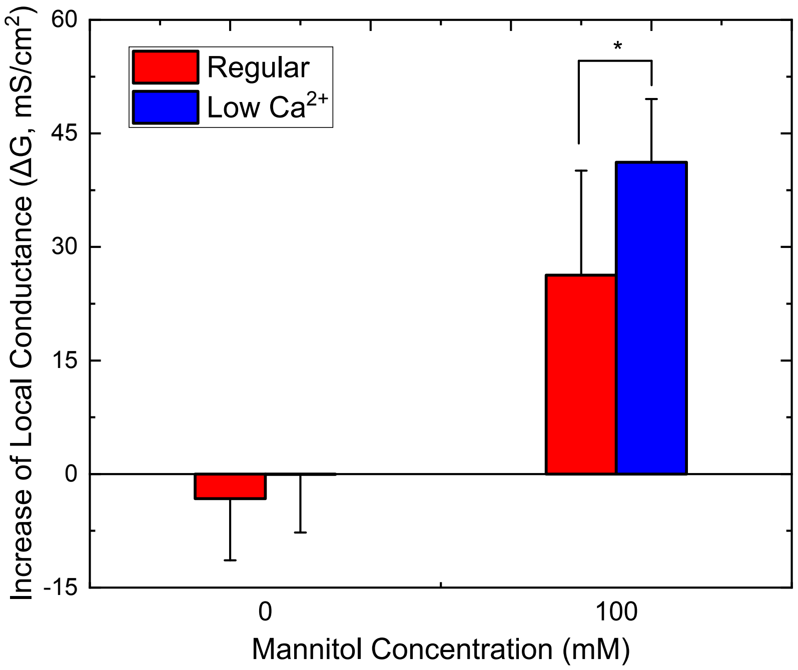

Figure S8b. The effect of Ca^{2+} concentration on the response of tTJs to hyperosmolality. (*$p < 0.05$, $n = 7$)

S6. The role of ILDR1 overexpression in hyperosmolar effect

The barrier properties of transfected ILDR1 cells were evaluated by EIS (Fig. S9). P-SICM measurements were then performed on ILDR1 cells with the same procedure and mannitol dosage as the wild type cells (Fig. S10 and Fig. 8).

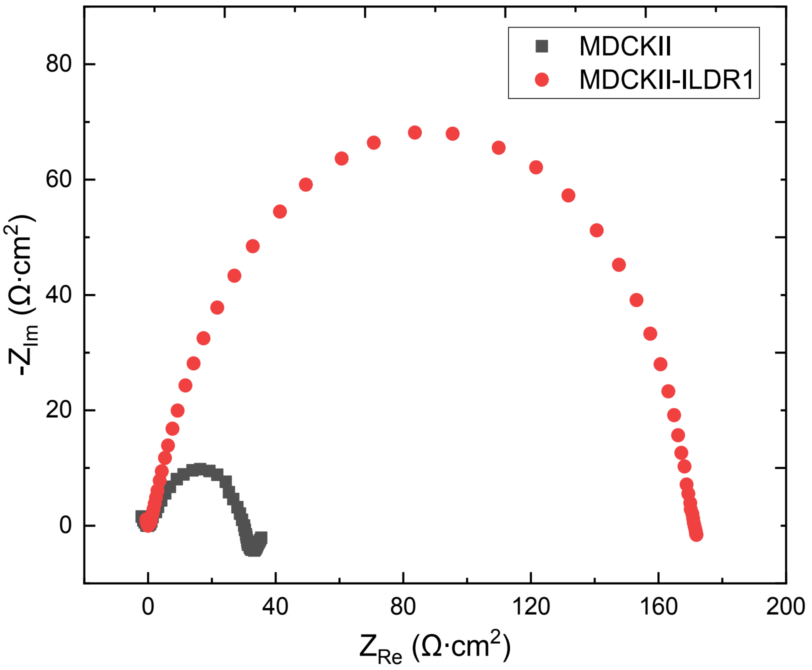

Figure S9. Representative impedance spectra of wild type MDCKII (black) and MDCKII-ILDR1 cells (red).

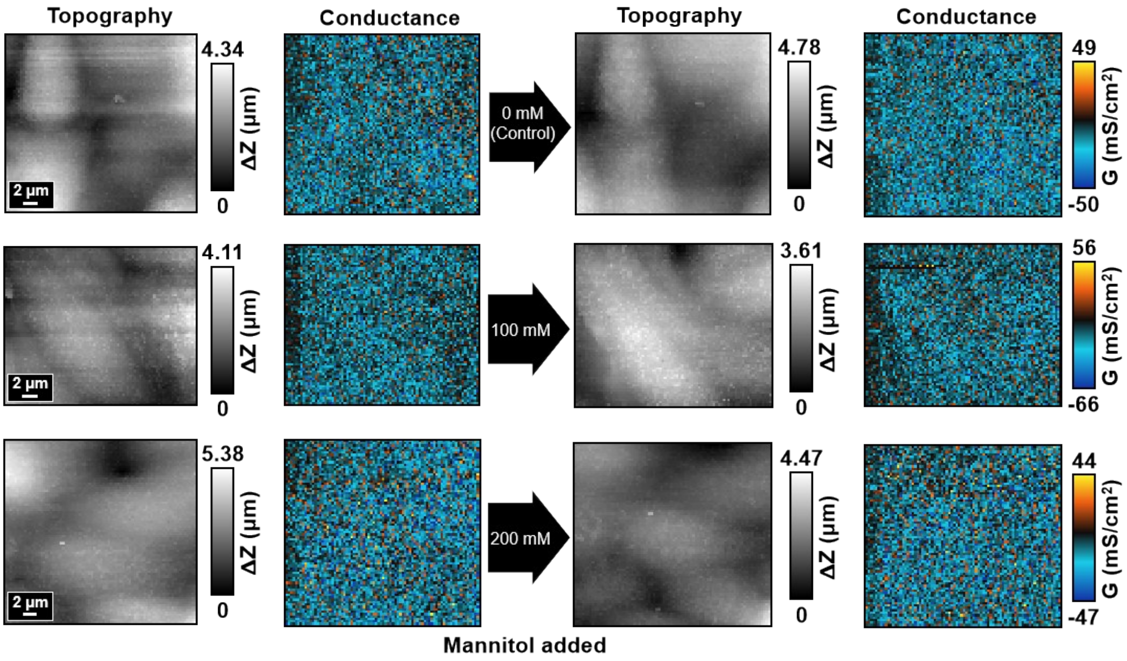

Figure S10. Topography and corresponding conductance maps of MDCKII-ILDR1 cells before (left) and after (right) mannitol treatment.

We modified the computer vision algorithm to extract the skeleton of junction area from the topography map of ILDR1 cells (Fig. S11). The input of the program is the 2D height data matrix of topography map. First, the height data are rescaled to [0,255] to create a grayscale uint8 image. Resulted uint8 image should appear almost identical to the original topography map. For each pixel of the image, it is compared to its 10 nearest neighbor pixels on both left and right side (in x direction). If the number of neighbors on one side is less than 10, all the neighbor pixels are taken into comparison. If the pixel is the smallest among its neighbors and itself, it is regarded as local minimal (or trough) point. The same process is conducted in y direction (i.e. comparison to neighbor pixels on top and bottom side). A binary image with identical size of the topography map is then generated, where the determined local minimal points are set as 1 (white) and the rest of pixels are set as 0 (black). The same image processing functions as ones used for conductance maps (i.e. remove incoherent non-zero pixels, hole closing, skeletonization and gap closer) can then be performed to the binary image as indicated in Fig. S11, from which the final skeleton image can be obtained.

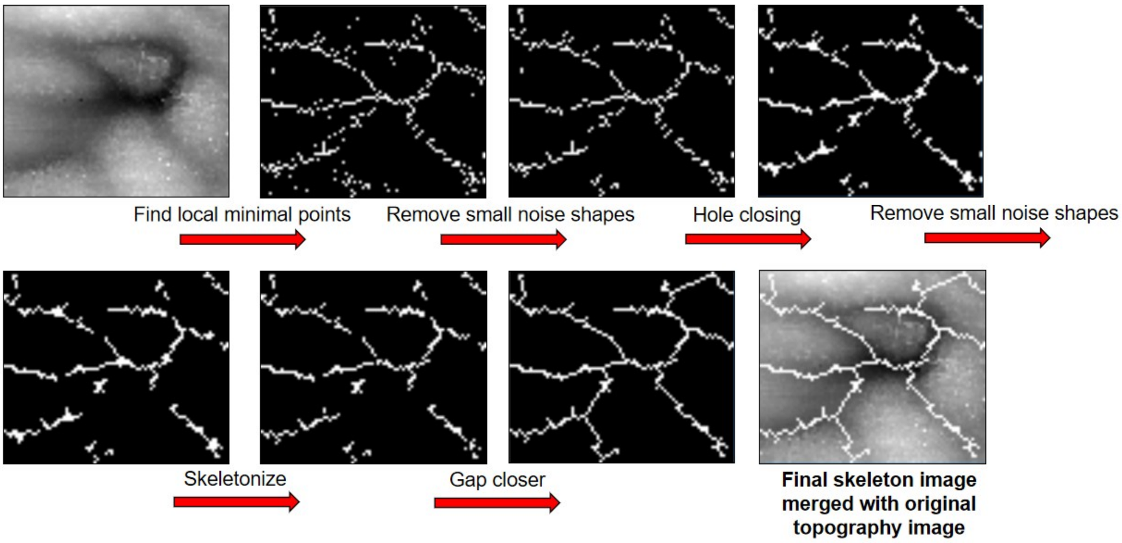

Figure S11. Schematic of computer vision algorithms finding bTJ and tTJ areas from topography maps of MDCKII-ILDR1 cells.

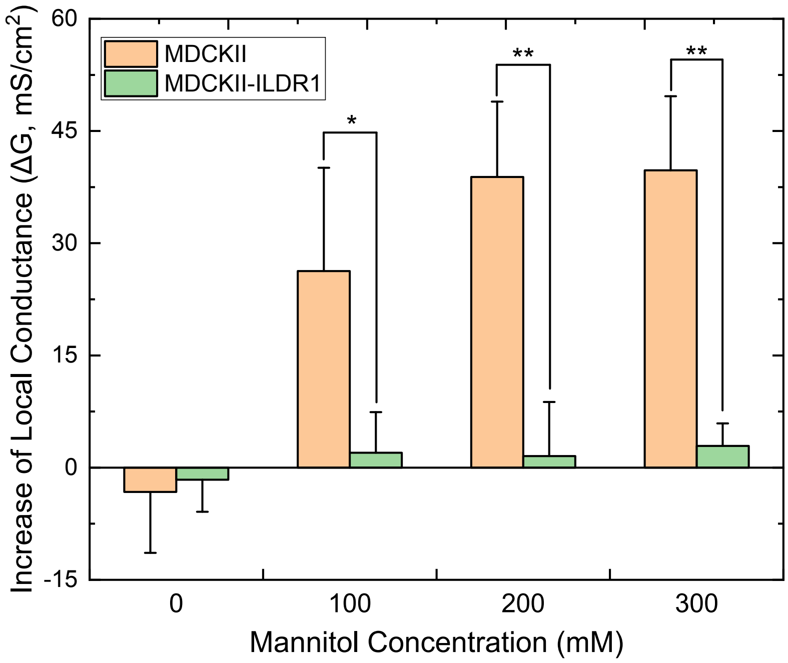

Figure S12. Comparison between MDCKII-WT and MDCKII-ILDR1 cells in the response of tTJs to hyperosmolality. (*$p < 0.05$, **$p < 0.01$, n = 6-9 for each condition)

References

1. Y. Gong, N. Himmerkus, A. Sunq, S. Milatz, C. Merkel, M. Bleich and J. Hou, *Proc. Natl. Acad. Sci. U.S.A.*, 2017, **114**, 5271.
2. L. Zhou, Y. Gong, J. Hou and L. A. Baker, *Anal. Chem.*, 2017, **89**, 13603-13609.
3. C.-C. Chen, Y. Zhou, C. A. Morris, J. Hou and L. A. Baker, *Anal. Chem.*, 2013, **85**, 3621-3628.
4. T. Y. Zhang and C. Y. Suen, *Commun. ACM*, 1984, **27**, 236-239.
